# A case report and literature review: an intra-abdominal epithelioid neoplasm with EWSR1::CREB fusions originating from the kidney

**DOI:** 10.3389/fonc.2025.1604933

**Published:** 2025-11-18

**Authors:** Zhen Zheng, Enjie Liu, Minglei Yang, Xiu Liu, Jianguo Wei

**Affiliations:** 1 Department of Pathology, Zhejiang Hospital, Hangzhou, China; 2 Department of Pathology, The First Affiliated Hospital of Zhengzhou University, Zhengzhou, China

**Keywords:** EWSR1::CREB fusion, intra-abdominal, epithelioid neoplasm, kidney, clinicopathological features

## Abstract

**Background:**

EWSR1::CREB fusion is a newly identified group of aggressive tumors with epithelioid morphology and multiple growth patterns. These tumors are often located in the abdominal cavity and frequently show cytokeratin expression immunohistochemically. This invasive epithelioid soft tissue tumor has a remarkable preference for mesothelial-lined cavities, with rare extension into intra-abdominal organs such as the kidney. Given its rarity, early diagnosis and treatment are crucial. Currently, the diagnosis and treatment of this disease pose significant challenges.

**Case demonstration:**

A 36-year-old male patient with no significant past medical history was admitted with a mass in the left kidney. Computed tomography showed a mass in the lower left kidney near the renal portal, and chromophobe carcinoma was suspected. The patient subsequently underwent a partial nephrectomy. The case was initially diagnosed as a malignant tumor with epithelial and mesenchymal components. RNA sequencing and FISH of the kidney mass confirmed the diagnosis of intra-abdominal epithelioid neoplasms with EWSR1::CREB fusions originating from the kidney. The patient did not undergo any adjuvant therapy and has been followed up for 14 months. He is currently in good condition.

**Conclusion:**

Intra-abdominal epithelioid neoplasm with EWSR1::CREB fusions originating from the kidney is rare. The remarkable morphological features of the case presented here further confirm the significant morphological heterogeneity of tumors characterized by EWSR1::CREB fusion and expand the morphological spectrum of malignant epithelioid tumors with EWSR1::CREM rearrangements originating from the kidney. Additionally, neoplastic cells encircled native renal tubules, demonstrating an infiltrating growth pattern, and the renal tubules proliferated significantly, which may lead to misdiagnosis as other tumors that exhibit biphasic morphology.

## Background

The EWSR1 gene encodes the RNA-binding protein EWSR1, which is a member of the TET family of transcription factors. These factors regulate various cellular processes like gene expression and RNA processing (1). There is increasing evidence that this gene is rearranged in various mesenchymal and epithelial tumors and can merge with many distinct genes (2). The fusion between EWSR1 and the CREB transcription factor family was particularly significant (3). CREB1, ATF1, and CREM form a subfamily of the alkaline leucine zipper (bZIP) superfamily of transcription factors, collectively known as the CREB family of transcription factors, that have multiple biological functions and regulate the expression of other genes (4). EWSR1 is a common fusion gene that fuses with CREB transcription factors, particularly ATF1 and CREB1, as 3′-partners (5). Furthermore, the fusion of the CREB gene family with EWSR1 or FUS gene partners leads to a wide variety of tumor pathogenesis. The EWSR1::CREB gene fusion was first described in a melanoma cell line (6). Later, EWSR1::CREB gene fusion was also reported in various mesenchymal tumors, including clear cell sarcoma of soft tissue, malignant gastrointestinal neuroectodermal tumor, hemangiomatoid fibrohistiocytoma, clear cell carcinoma of the salivary gland, clear cell odontogenic tumor, myoepithelial tumor, primary pulmonary myxoid sarcoma and primary intracranial myxoid sarcoma, paraganglioma, malignant mesothelioma in young adults, and intra-abdominal epithelioid malignancies. Masato et al. (5) statistically found that EWSR1/FUS::CREB rearranged tumors could occur in diverse anatomical locations, including the brain, soft tissue, head and neck, lung, internal organs, and abdominal cavity. Currently, EWSR1/FUS::CREB rearranged epithelioid malignancies have been documented in various intra-abdominal locations, such as the adrenal glands, colon, kidneys, liver, pancreas, stomach, and uterine adnexa (7).

## Case demonstration

A 36-year-old male patient was referred in 2024 to the Department of Urology with a finding of a mass on the left kidney for 7 days. He had been diagnosed with a cyst in the left renal parenchyma area 1 year earlier during physical examination, but he had no further tests performed at that time. During physical examination in July 2024, ultrasound revealed once more a hypoechoic area in the left kidney, and further examination was recommended. The patient underwent computed tomography (CT) imaging at another unit, and a mass in the lower pole of the left kidney was found. The possibility of chromophobe renal cell carcinoma was considered.

The patient had no past medical history of hypertension or heart disease. He had no family history of cancer or hereditary diseases that were known. Moreover, he was a non-smoker and a non-drinker.

**Figure 1:**
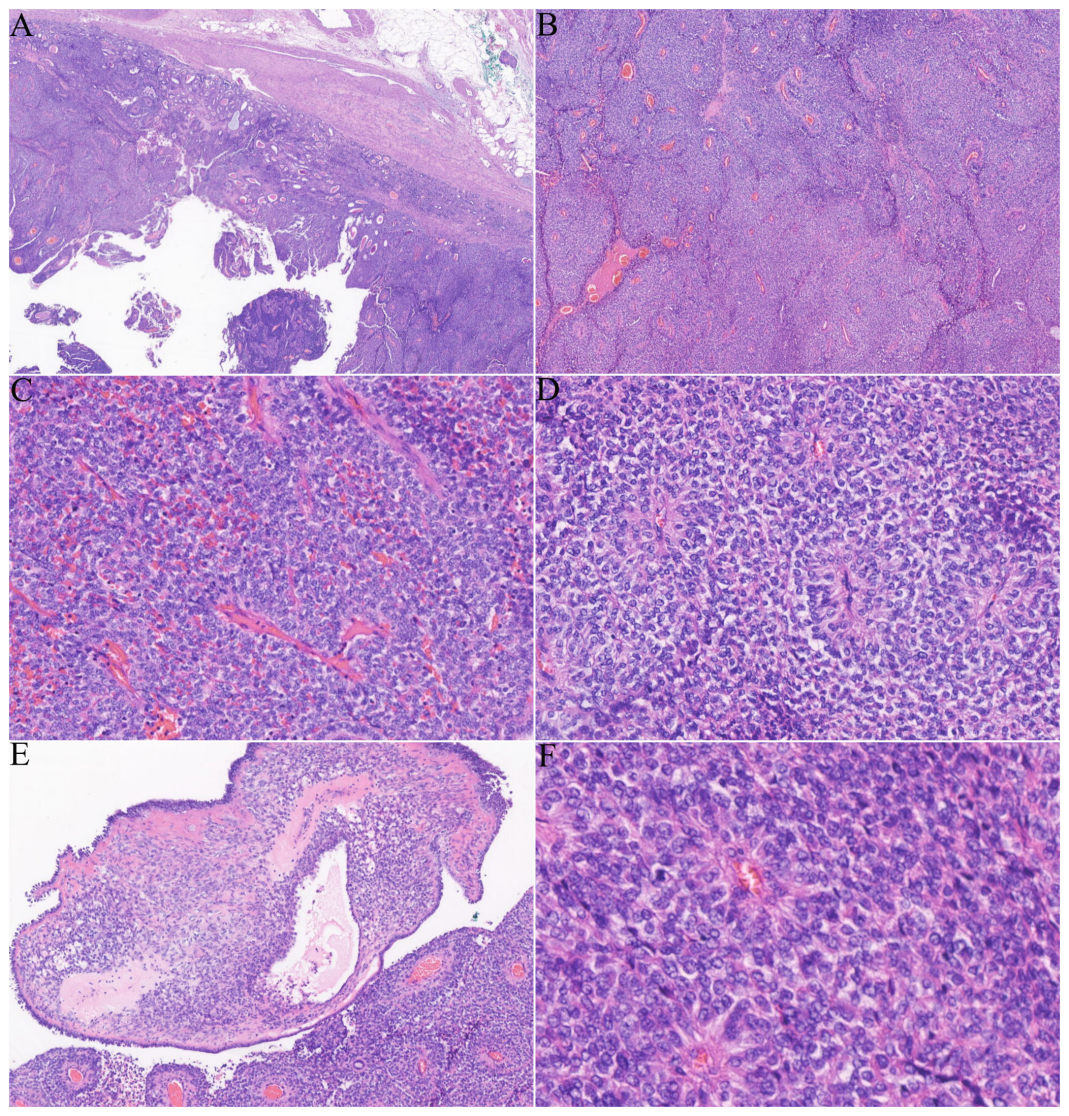
Histological features of the tumor: **(A)** a tumor with clearly defined edges and a fibrous envelope. **(B, C)** The solid and nested tumor cells displayed a hemangiopericytomatoid growth pattern. **(D)** Dense tumor cells formed clusters resembling pseudochrysanthemums around blood vessels. **(E)** Localized microcapsules contained pink liquid. **(F)** The cell boundaries were unclear, with round or oval nuclei and low mitotic activity (1–2/10 HPFs).

**Figure 2:**
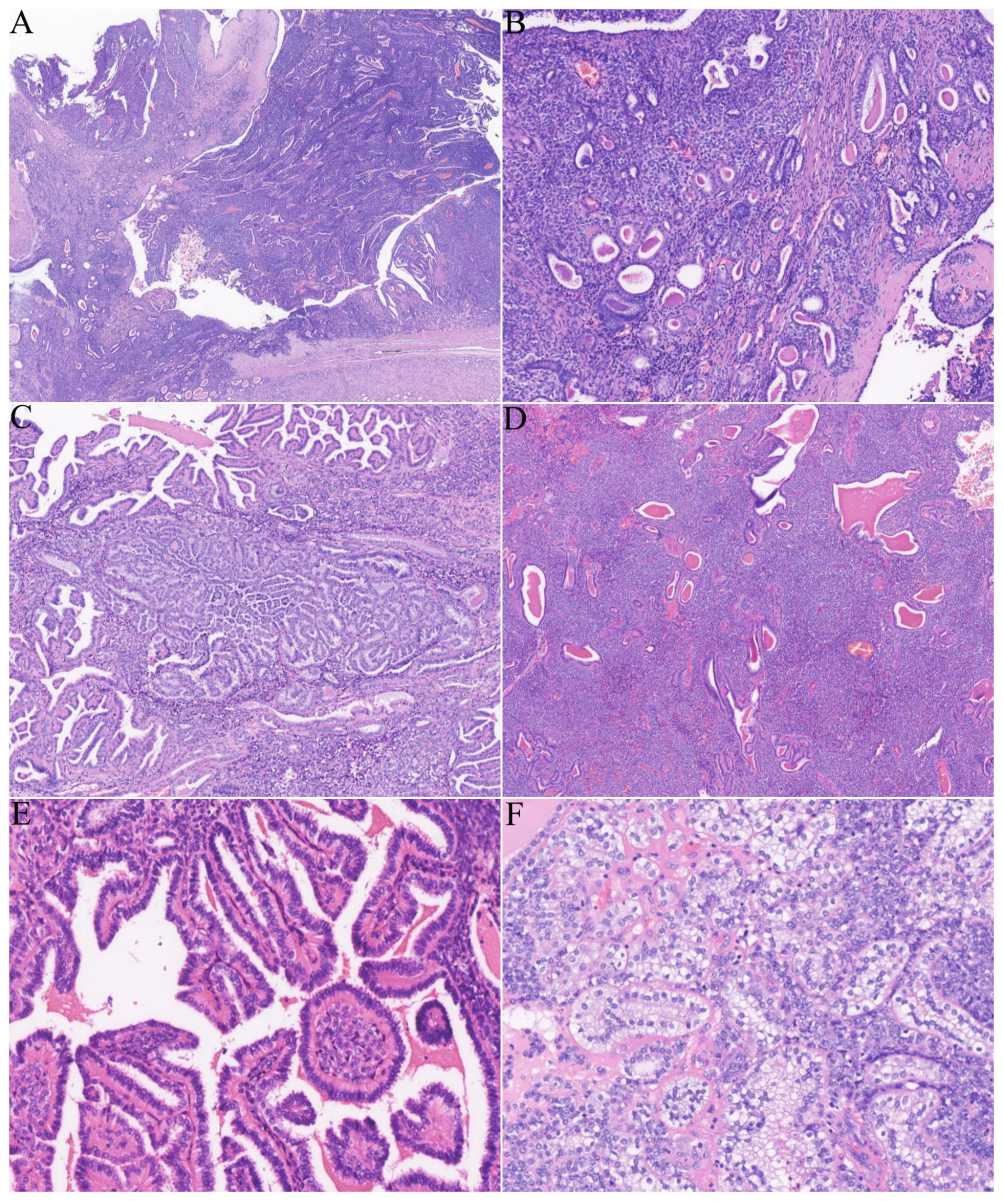
The tumor involved abundant renal tubules, particularly in the surrounding areas. **(A)** Low magnification revealed numerous renal tubules involved both around and within the tumor. **(B)** Small tubular and annular structures. **(C)** The affected renal tubules showed significant proliferation, resulting in the formation of micropapillae, papillae, and cribriform structures. **(D)** Some renal tubules displayed either slit-like or cystic dilatation, accompanied by eosinophilic secretions found in the lumen. **(E)** Renal tubular epithelial cells are mild, unilinear, featuring rounded nuclei and eosinophilic cytoplasm. **(F)** Certain areas of proliferative tubular epithelial cells contain a clear cytoplasm.

The patient subsequently underwent a partial nephrectomy at the First Affiliated Hospital of Zhengzhou University. Grossly, the tumor measured 5.5 cm × 5.0 cm × 2.3 cm. On microscopic examination, the tumor was well circumscribed, surrounded by a fibrous capsule (**Figure 1A**). The neoplastic cells were arranged in nests and sheets (**Figure 1B**) around the blood vessels, forming a hemangiopericytomatoid growth pattern (**Figure 1C**) in concordance with what was reported by Zhao et al. (1): a pseudochrysanthemum-like structure (**Figure 1D**), with local cystic or microcystic changes (**Figure 1E**). Round or short fusiform tumor cells displayed monomorphic nuclei with smooth nuclear contours and open chromatin. Tumor cells showed a slightly eosinophilic or transparent cytoplasm, with variably prominent nucleoli. The tumor showed relatively low mitotic activity (1 to 2/10 HPFs) and lacked nuclear pleomorphism (**Figure 1F**). The interstitial fibrocollagen was not prominent, and there were no signs of inflammatory cell infiltration or necrosis. Foam-like histiocyte aggregation, multinucleated giant cell responses, and cholesterol crystallization deposits were focally seen. In particular, neoplasms encircled a higher number of native renal tubules, which were especially pronounced around the tumor. The markedly hyperplastic renal tubules (**Figure 2A**) exhibited tubular and annular shapes (**Figure 2B**), with certain areas characterized by the presence of nipples, micronipples, and complex cribriform structures (**Figure 2C**). Some lumens were either dilated or slit-like, containing eosinophilic secretions (**Figure 2D**). The renal tubular epithelial cells were arranged in a single row, closely aligned focally, with overlapping nuclei. The epithelium cells showed mild morphological features, such as round nuclei, clear nuclear membranes, fine chromatin, and detectable nucleoli, while showing no mitoses (**Figure 2E**). The cytoplasm was eosinophilic and partially transparent (**Figure 2F**).

**Figure 3:**
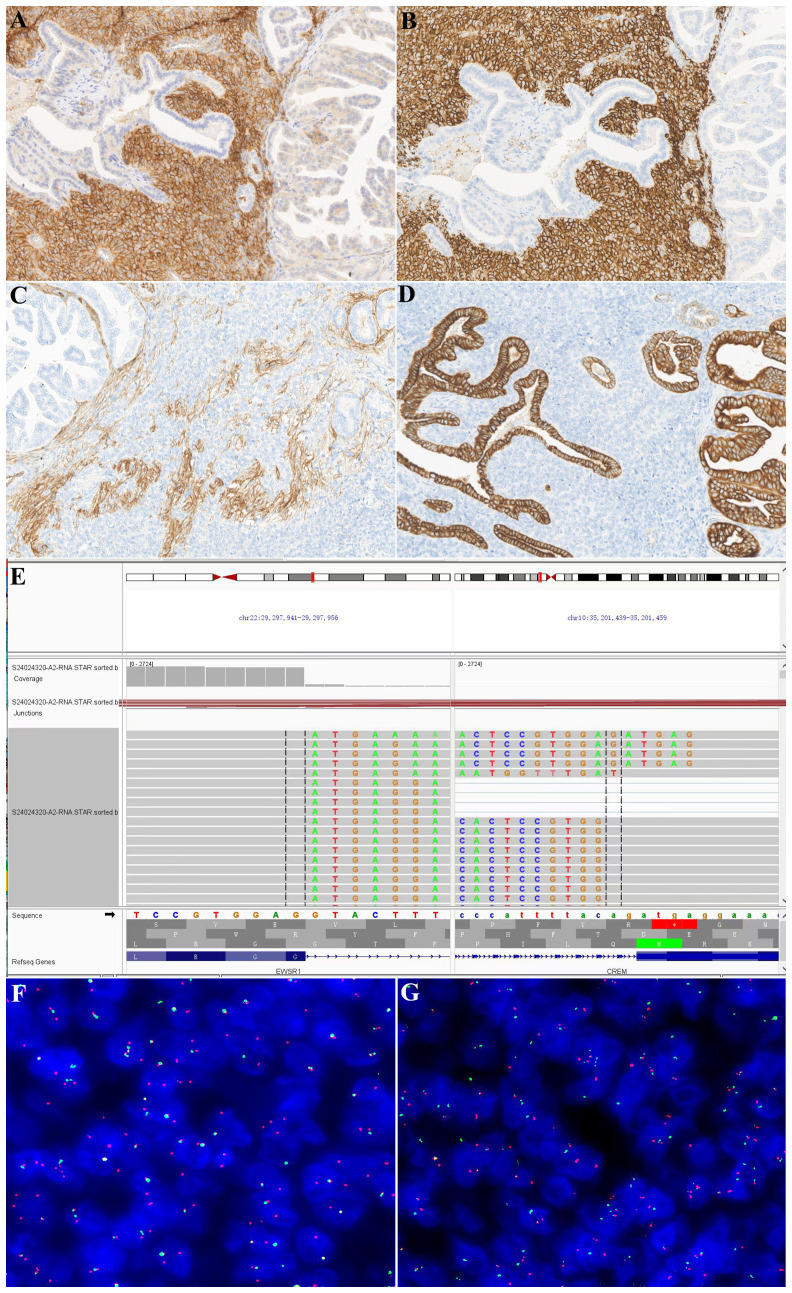
Immunohistochemical and molecular findings. This neoplasm was diffusely positive for CD99 **(A)** and CD56 **(B)**, and the affected tubular epithelium was negative. The tumor cells showed focal positivity for CD34 **(C)** but were negative for AE1/AE3 **(D)**. In contrast, the affected tubular epithelium was negative for CD34 and diffusely positive for AE1/AE3. RNA sequencing showed EWSR1::CREM fusions **(E)**. FISH confirmed the results of RNA sequencing [**(F)** EWSR1 broken signals; **(G)** EWSR1 and CREM fusion signals].

Immunohistochemical studies revealed that the neoplastic cells were diffusely positive for CD99 (**Figure 3A**), CD56 (**Figure 3B**), and CD57, while the involved renal tubules were not immunoreactive. Cyclin D1 showed a moderately positive result. SMA and CD34 were focally positive (**Figure 3C**). AE1/AE3 (**Figure 3D**), CK7, and PAX8 were negative in the tumor cells, whereas the adjacent tubular epithelial cells showed diffuse and strong positivity. Additionally, CK20, P504s, WT-1, P63, GATA 3, and renin were negative in the tumor cells. ALK (5A4) and syn showed partial positivity, and the Ki-67 proliferation index was approximately 10%. RNA sequencing revealed EWSR1::CREM gene fusion (**Figure 3E**). FISH analysis confirmed the EWSR1 break (**Figure 3F**) and EWSR1::CREM fusion (**Figure 3G**), whereas no BRAF V600E gene alteration was detected by fluorescence PCR.

Based on the clinical history, imaging features, histological morphology, immunohistochemical results, and molecular results, the patient was pathologically diagnosed with intra-abdominal epithelioid neoplasm with EWSR1::CREB fusions originating from the kidney. No local recurrence or metastatic lesions were detected 14 months after the surgery without adjuvant therapy, and he is currently in good condition.

## Discussion

With the widespread use of RNA sequencing technology, the EWSR1/FUS::CREB fusion mesenchymal tumor family has expanded rapidly to include potentially aggressive tumors rather than any well-established WHO entity. EWSR1/FUS::CREB fusion has been considered to define a group of aggressive tumors with epithelioid morphology. Primary tumors presenting as intraperitoneal internal organs are rare and involve the mesothelial space, often forming a mass in the abdominal cavity (8).

Including our case, there has been a total of nine cases (4, 8–11) of intraperitoneal malignant epithelioid tumors with EWSR1/FUS::CREM rearrangement that originated in the kidney described in the literature. Clinical and pathologic characteristics are summarized in **Table 1**. Of the nine tumors, five were found in female patients and four in male patients; these patients had a median age of 34 years (range, 17 to 61 years) at diagnosis, whose tumors ranged from 4.0 to 30.0 cm (median: 7.5). All patients presented with a mass in the kidney, with four and three tumors affecting the left and right kidney, respectively. The locations of the other two tumors were not specified. Seven patients underwent radical nephrectomy, one patient had a left renal tumor resection, while the procedure for the rest of the patients was unknown. Six patients had no adjuvant therapy performed after surgery, while two patients underwent adjuvant chemotherapy (10, 11).

**Table 1:** Clinicopathological and molecular characteristics of intraperitoneal malignant epithelioid tumors with EWSR1::CREB rearrangement originating from the kidney.

Case	Age/sex	Size (cm)	Site	Therapy	Histology	RNA sequencing	FISH	Clinical follow-up
Zhao et al. (4)	18/F	7.8	Left	Radical nephrectomy, NDT	SEF-like, with hyaline stroma	EWSR1(ex4)::CREM(ex7) fusion	EWSR1::CREM fusion	5 months, multiple bone and spinal metastases
	17/M	30.0	Right	Radical nephrectomy, NDT	SEF-like, with hyaline stroma	EWSR1(ex16)::CREM(ex5) fusion	EWSR1::CREM fusion	9 months, NED
	61/F	5.6	Left	Radical nephrectomy, NDT	SEF-like, with hyaline stroma	EWSR1(ex4)::ATF1(ex4) fusion	EWSR1::ATF1 fusion	120 months, NED
Agaimy et al. (9)	55/M	7.5	Unknown	Radical nephrectomy, NDT	Epithelioid cells and fibrous stroma	EWSR1::CREM fusion	EWSR1::CREM fusion	23 months, pelvic metastases
Agaimy et al. (10)	34/F	7.5	Right	Radical nephrectomy, Seven cycles of adjuvant chemotherapy	Epithelioid or round cell composition	EWSR1(ex 16)::CREM (ex 6) fusion	Not done	50 months, NED
Argani et al. (8)	29/M	10.0	Unknown	Unknown	Cystic solid; tumor cells were round and epithelioid	EWSR1::CREM fusion	Not done	Peritoneum, para-aortic metastasis
Li et al. (11)	31/F	5.4	Left	Radical nephrectomy, adjuvant chemotherapy	Round and epithelioid present	EWSR1::CREM	Not done	21 months, NED
	38/F	4.0	Right	Radical nephrectomy, NDT	Round and epithelioid	Not done	EWSR1 isolation and EWSR1::CREB1 fusion	33 months, NED
This case	36/M	5.5	Left	Partial nephrectomy, NDT	Tumor cells were round or short fusiform, showing renal tubular involvement and significant proliferation	EWSR1::CREM fusion	EWSR1::CREM fusion	14 months, NED

F, female; male; SEF, sclerosing epithelioid fibrosarcomatoid; NDT, no adjuvant therapy was performed; NED, no evidence of disease.

Histologically, three tumors were not encapsulated and displayed irregular borders, while the other six tumors displayed clear boundaries with surrounding renal tissue, four of which had a visible fibrous pseudocapsule. Argani et al. (8) and Wu et al. (12) have outlined the key morphological features of intraperitoneal malignant epithelioid tumors with EWSR1/FUS::CREM rearrangement as follows: i) the presence of epithelioid cells, which can be mixed with epithelial/spindle cells or epithelial/round cells; ii) cystic or microcystic changes; and iii) varying amounts of chronic inflammatory cell infiltration. Among the nine patients, seven tumors exhibited round epithelioid cells, while one tumor was composed of round cells alongside scattered epithelioid cells. The current renal neoplasm, characterized by round and short fusiform shapes, showed cytoplasmic transparent epithelioid cells in the focal area, which is reported for the first time in the kidney. Cystic or microcystic changes were noted in four tumors. Three tumors showed prominent intratumoral inflammation, not including the current tumor. The mitotic activity ranged from 1 to 20/10 HPFs (mean, 4), and necrosis was present in four cases. Notably, neoplasms encircled native renal tubules, and/or a glomeruli growth pattern was commonly seen in four cases, especially in our case, where the involved renal tubular tissue showed prominent hyperplasia displaying a variety of morphological structures, which had not previously been emphasized in EWSR1::CREB fusion neoplasms involving the kidney. Other patterns that were noted in most cases included hemangiopericytoma-like structures, pseudochrysanthemum cluster structures, collagenous sclerotic interstitium, rhabdomyoid cells, and pseudoalveolar-like structures. Additionally, we also observed local histiocytic aggregation, cholesterol crystallization, and a multinucleated giant cell reaction. Immunohistochemically, the reported eight neoplasms showed variable expression of epithelial markers (AE1/AE3, EMA), six tumors showed MUC4 expression, and four cases were immunoreactive for CD99. Our case showed CD56 and CD57 positivity, while AE1/AE3 was negative. This is the second case of an AE1/AE3-negative condition. In the other case of AE1/AE3 negativity, EMA was focally positive (11). Seven neoplasms showed EWSR1::CREM fusion by RNA-seq, and four tumors were confirmed by FISH. Additionally, one neoplasm exhibited EWSR1::ATF1 fusion (4), and another one exhibited EWSR1::CREB1 fusion (11).

According to previous literature, intra-abdominal epithelioid neoplasms with EWSR1::CREB fusions originating from the kidney should be considered in the differential diagnosis of sclerosing epithelioid fibrosarcoma (SEF), angiomatoid fibrous histiocytoma (AFH), epithelioid mesothelioma (EM), clear cell sarcoma of the kidney (KCCS), synovial sarcoma (SS), solitary fibrous tumors (SFT), or metanephric stromal tumor (MST) (4). The key points for identification are listed in **Table 2**. From a morphologic perspective, the most important distinction to be made in our case is bidirectional synovial sarcoma (BSS). The cases presented here showed a large amount of normal renal tubular tissue wrapped by tumor tissue, with tubular, annular, papillary, micropapillary, or complex cribriform structures forming biphasic morphology with short spindle tumor cells, mimicking SS. SS occurs mainly in the deep soft tissues of the extremities and is rarely seen in the kidney. Biphasic SS comprises different proportions of epithelioid cells and spindle cells. Epithelioid cells may contain glandular secretion or mucus, and they may form papillary, beam, and solid nest mass patterns. More than 95% of SS has the characteristic t(X; 18) (p11.2; q11.2) chromosomal translocations resulting in the generation of SS18::SSX gene fusion, subsequently resulting in diffuse, positive nuclear expression of SS18-SSX (13). The other tumor to be differentiated from our case is renal carcinosarcoma (RC). Renal carcinosarcoma is a highly malignant tumor that occurs in the kidney and has both epithelial and mesenchymal differentiation (14). The malignant epithelial component originates from the renal tubular epithelium or renal pelvis epithelium, and the pathological manifestations are renal cell carcinoma and transitional cell carcinoma. The sarcomatous components are derived from the renal interstitium, and the pathological manifestations are fibrosarcoma, leiomyosarcoma, and other mesenchymal tissue sarcomas. Finally, given the characteristic features of extensive infiltrating growth pattern that result from its interaction with entrapped native renal elements, as well as the marked hyperplasia of renal tubules, the differential diagnoses should also include an MST, which is an extremely rare benign tumor of the kidney. The tumor cells are spindle-shaped or stellate, and they are arranged characteristically in a “concentric circle” pattern around the invaginated blood vessels or renal tubules, resembling an “onion skin-like change,” with dysplasia of the invaginated blood vessels. Immunohistochemistry reveals that the tumor cells exhibit varying degrees of positive CD34 expression. The BRAF V600E mutation is frequently present (15). In this case, the BRAF V600E gene alteration was not detected by fluorescence PCR, thereby ruling out MST. For a renal epithelial-like tumor, the following diagnostic process is recommended: First, morphological screening should be conducted to determine whether the tumor is mainly composed of epithelial-like cells or contains other components (such as spindle cells, glands, and heterologous components). Second, immunohistochemical screening can be performed using a set of immunohistochemical markers such as PAX8, CK, CD99, CD34, ERG, STAT6, MUC4, SS18-SSX, BCOR, and WT1. If all the above markers are negative or only CK is positive (including diffuse positivity or focal positivity), EWSR1::CREB fusion epithelioid tumors, angiomatoid fibrous histiocytomas, and other conditions should be suspected. The final diagnosis must rely on molecular testing, such as FISH detection (EWSR1 break probe) or next-generation sequencing, to determine whether there is EWSR1::CREB family fusion and to pay attention to the final distinction from a morphologically similar angiomatoid fibrous histiocytoma. In summary, an EWSR1::CREB fusion epithelial-like tumor originating from the kidney is an “exclusively” diagnosable condition. A systematic morphological, immunohistochemical, and molecular pathological analysis is required to rule out all the above similar tumors before making an accurate diagnosis.

**Table 2:** Differential diagnosis of intraperitoneal malignant epithelioid tumors with EWSR1::CREB rearrangement originating from the kidney.

Tumor	Key morphological features	Core immunohistochemical markers	Genetic alteration
Intra-abdominal epithelioid neoplasm with EWSR1::CREB fusions	Epithelioid cells are dominant, with rare true spindle cell components and mucoid or collagenous stroma.	AE1/AE3 and EMA were focal or dot-like or had diffuse positivity, whereas PAX8 and WT1 were negative.	EWSR1::CREM/ATF1, EWSR1::CREB1 (common)
BSS	The tumor exhibits a distinct biphasic pattern composed of spindle and epithelioid cells, with the formation of glandular lumina and potential stromal calcification.	EMA and CK (AE1/AE3) showed strong positivity in the epithelial area. TLE1 was nuclear positive (sensitive but not specific). Simultaneous positivity for S100 and CD99 is helpful for diagnosis. The positivity of SS18-SSX antibodies is related to specificity.	t(X;18), resulting in the SS18::SSX1/2/4 fusion gene
RC	For the vast majority of carcinosarcomas, if sufficient tissue samples are obtained, different proportions/types of renal cell carcinomas can be observed.	The cancerous area expresses markers such as PAX8, PAX2, and CK. The sarcoma area expresses corresponding mesenchymal or epithelial markers to varying degrees (such as SMA, desmin, S100).	The molecular genetics of different types of renal cell carcinomas vary considerably. For instance, clear cell carcinomas may have a 3-chromosome deletion or be associated with VHL syndrome, while papillary renal cell carcinomas may have trisomy of chromosomes 7 and 17 and deletion of the Y chromosome.
MST	The cells are mainly in a spindle shape, forming a “sock-like” structure that surrounds the renal tubules. The background is composed of collagen. This condition is more common in children.	CD34+, WT1−, S100−, PAX8−	The BRAF V600E gene mutation is a key molecular alteration
SEF	Epithelioid and spindle cells are embedded in dense sclerotic collagen, with a low cell density.	MUC4 showed a strong positive reaction (highly sensitive and specific), and almost all markers mentioned in this article were negative.	The most common gene rearrangement is EWSR1::CREB3L1, with a few being EWSR1::CREB3L2 fusion
AFH	Multiple nodular formations, pseudo-vascular tumor-like cavities, and lymphocytic cuffs. The tumor cells can be spindle-shaped, histiocyte-like, or epithelioid.	Desmin+ (50%), EMA+ (40%), and CD99 may be positive.	EWSR1::CREB fusion, or there can also be FUS::ATF1 fusion.
EM	The epithelioid cells are arranged in sheet-like, tubular, and papillary patterns, and the cytoplasm is often eosinophilic and basophilic.	CK5/6+, calretinin+, WT1+ (nuclear+), D2-40+, PAX8−	BAP1 deficiency (common), CDKN2A homozygous deletion
CCSK	The morphology is diverse, the nucleolus is not prominent, the cytoplasm is lightly stained, and the interstitium is rich in branched vascular networks. This is commonly seen in children.	BCOR+, cyclin D1+, WT1−, PAX8−	BCOR : CCNB3 fusion, BCOR ITD or YWHAE::NUTM2
SFT	“Unstructured” pattern, spindle-shaped cells, antler-shaped blood vessels, and collagenization.	STAT6+ (nuclear), CD34+	NAB2::STAT6 fusion

BSS, bidirectional synovial sarcoma; RC, renal carcinosarcoma; MST, metanephric stromal tumor; SEF, sclerosing epithelioid fibrosarcoma; AFH, angiomatoid fibrous histiocytoma; EM, epithelioid mesothelioma; CCSK, clear cell sarcoma of the kidney; SFT, solitary fibrous tumor.

Currently, no standard treatment exists for neoplasms with EWSR1::CREB fusions (6), while complete surgical resection with negative margins is recommended as the main treatment strategy for these genetically defined tumor types (16). According to the reports in earlier literature, most patients underwent radical nephrectomy (seven of nine) (4, 8–11), while our case underwent partial nephrectomy but with negative margins. In addition, only two patients (10, 11) received adjuvant chemotherapy after surgery. One patient (10) underwent seven consecutive cycles of chemotherapy. Most patients (six of nine) had a good prognosis with no signs of disease for 5 to 120 months after the initial diagnosis, while three patients experienced metastases during follow-up, including multiple bone and spinal lesions (4), pelvic (9), and peritoneal and para-aortic (8) metastases. Overall, three of nine tumors were progressive at the last follow-up, indicating an overall progressive course in 33.3% of patients, indicating the highly aggressive nature of the tumor. Therefore, such tumors may require long-term follow-up to monitor the possibility of late recurrence or metastasis. In addition, large-scale reports of tumors with EWSR1/FUS::CREB fusion features, whether intrarenal or extrarenal, are rare in the literature. A relatively large number of cases were reported by Argani et al. (8) and Shibayama et al. (17), with 13 and 8 cases of tumors with EWSR1/FUS::CREB fusion, respectively. A total of 21 cases were included, comprising 8 with EWSR1::CREM fusion, 8 with FUS::CREM fusion, 4 with EWSR1::ATF1 fusion, and 1 with EWSR1::CREB1 fusion. Among patients with EWSR1::CREM fusion, the maximum follow-up duration was 204 months. Of these, five cases (5 of 8) experienced recurrence or metastasis, one case succumbed to the disease, and one case remained free of recurrence or metastasis during follow-up. Four patients with FUS::CREM fusion were lost to follow-up, while the remaining four patients had the longest follow-up period of 58 months. Three patients experienced recurrence or metastasis (3 of 4), among whom two died of tumors, and one had no recurrence or metastasis during the follow-up period. Two patients with EWSR1::ATF1 fusion experienced recurrence or metastasis during the follow-up period, of which one died of the tumor, and two had no recurrence or metastasis during the follow-up period. The longest follow-up period was 25 months. There was only one patient with EWSR1::CREB1 fusion. The follow-up duration for this patient was 122 months; however, there were two recurrences. The recurrence or metastasis rates of the four different fusion genes were 62.5%, 75.0%, 50.0%, and 100%, respectively, and the mortality rates were 12.5%, 50.0%, 25.0%, and 0%, respectively. Based on these data, the EWSR1::CREB1 fusion had the highest recurrence or metastasis rate, followed by the FUS::CREM fusion. The FUS::CREM fusion had the highest mortality rate, followed by the EWSR1::ATF1 fusion. However, these data are limited by the small number of cases and require more data support.

## Conclusion

In summary, we describe a new case of intra-abdominal epithelioid neoplasm with recurrent EWSR1::CREB gene fusions involving the kidney, which exhibited notable native renal tubular hyperplasia and were immunonegative for keratin AE1/AE3. The unusual histological morphology and immunophenotype have not been previously reported in the literature, which could lead to misdiagnosis as tumors with bidirectional morphology, such as synovial sarcoma and carcinosarcoma. Molecular detection, along with morphological and immunohistochemical observations, is essential for accurately diagnosing this type of tumor.

## Data Availability

The original contributions presented in the study are included in the article, further inquiries can be directed to the corresponding author.
